# Effects of musical training and event probabilities on encoding of complex tone patterns

**DOI:** 10.1186/1471-2202-14-51

**Published:** 2013-04-24

**Authors:** Anja Kuchenbuch, Evangelos Paraskevopoulos, Sibylle C Herholz, Christo Pantev

**Affiliations:** 1 Institute for Biomagnetism and Biosignalanalysis, University of Münster, Münster, Germany; 2 Montreal Neurological Institute, McGill University, Montreal, Quebec, Canada; 3 German Center for Neurodegenerative Diseases (DZNE), Bonn, Germany

**Keywords:** Processing of tone patterns, Mismatch negativity, Musical training, MEG

## Abstract

**Background:**

The human auditory cortex automatically encodes acoustic input from the environment and differentiates regular sound patterns from deviant ones in order to identify important, irregular events. The Mismatch Negativity (MMN) response is a neuronal marker for the detection of sounds that are unexpected, based on the encoded regularities. It is also elicited by violations of more complex regularities and musical expertise has been shown to have an effect on the processing of complex regularities. Using magnetoencephalography (MEG), we investigated the MMN response to salient or less salient deviants by varying the standard probability (70%, 50% and 35%) of a pattern oddball paradigm. To study the effects of musical expertise in the encoding of the patterns, we compared the responses of a group of non-musicians to those of musicians.

**Results:**

We observed significant MMN in all conditions, including the least salient condition (35% standards), in response to violations of the predominant tone pattern for both groups. The amplitude of MMN from the right hemisphere was influenced by the standard probability. This effect was modulated by long-term musical training: standard probability changes influenced MMN amplitude in the group of non-musicians only.

**Conclusion:**

This study indicates that pattern violations are detected automatically, even if they are of very low salience, both in non-musicians and musicians, with salience having a stronger impact on processing in the right hemisphere of non-musicians. Long-term musical training influences this encoding, in that non-musicians benefit to a greater extent from a good signal-to-noise ratio (i.e. high probability of the standard pattern), while musicians are less dependent on the salience of an acoustic environment.

## Background

The capacity for detecting unexpected and possibly important or dangerous events is essential for adaptive behaviour. The human auditory cortex automatically encodes acoustic input from the environment and differentiates regular sound patterns from deviant ones in order to identify possibly important, irregular events, such as a sudden change in the sound of a car that might indicate engine malfunction.

The Mismatch Negativity (MMN) response is a marker for the detection of sounds that are unexpected given a basis of previously encoded regularities. It has been widely used to investigate the processing of auditory stimuli in the auditory cortex in healthy and clinical populations [1-5]. MMN-like responses have also been reported in animals [6-8], which have further elucidated the physiological basis of the MMN response.

The MMN is elicited not only by violations of simple acoustic features or local-features, such as pitch or timbre, but also by violations of more complex or global regularities. To detect violations of local regularities, a current auditory event has to be compared constantly to an established regularity. The comparator mechanism underlying the MMN has therefore been described as a memory-based process and the temporal window of integration (TWI) of echoic memory is typically assumed to be around 10s [9]. There is evidence that global regularities, rather than local regularities, can also determine whether a tone is perceived as a violation and can therefore elicit an MMN response [10-13]. For example, a pattern regularity like “W” is followed by tone “Y” is followed by tone “Z” is not based on the repetition of one repeatedly presented tone but instead on the sequence of 3 different tones which have to be grouped in order to establish the pattern regularity. If the pattern is more complex and is additionally presented at a rather low frequency, the establishment of the pattern regularity exceeds the TWI and cannot be based on local regularities, but rather rely on higher-order memory systems that can integrate over a longer time frame than 10s. Herholz and colleagues, for example, found that expectancies of tones in a continuous tone stream are established by global regularities (tone patterns) and that the MMN elicited in response to violations of the established regularity is based on global statistical knowledge rather than a local memory span. In conclusion, the MMN represents a more general violation detection mechanism that does not necessarily need to be based on local regularities. The elicited MMN could be found in response to violations of the predominant standard pattern occurring with a probability of only 50% [12].

Evidence from an animal study supports this idea: a single-neuron phenomenon, a decrease in the response to a repeated stimulus, which does not generalize to other stimuli, called stimulus-specific adaption (SSA) has been associated with MMN. Studying neurons in the primary auditory cortex of cats, Ulanovsky and colleagues (2004) found that responses to tone-pattern-like sequences of tones A and B (for example BBA, ABA) depend upon the overall stimulus probability: by decreasing deviant probability (10%-90% deviant probability), and therefore increasing standard probability, the neuronal response increases [14]. Other studies on SSA in rats and rodents show that SSA is a different process than the human MMN, since neurons do not generate a late deviant response component directly equivalent to the human MMN [15,16]. Recent investigations by Sculthorpe and Campbell (2011) into the way in which the MMN response in tone patterns is influenced by different rare deviant probabilities (0.02 to 0.16% deviant probability) of the violating event, and thereby different probabilities of the standard event, concluded that the MMN amplitude does not vary with deviant probability [17] under these conditions. All deviant probabilities were, however, very low (and the standard probabilities, accordingly, very high) compared to the wider range of event probabilities used in the experiment of Ulanovsky et al. (2004). Various studies show that simple feature MMN is influenced by standard probability, through an inverse relationship between MMN amplitude and deviant probability [18-22]. The lower threshold of standard probability that is required for the successful encoding within the auditory cortex of an auditory pattern (standard probability) among other tones in an acoustically variable environment is, however, unknown.

Expertise shapes brain anatomy and brain physiology. Therefore experts often show greater abilities within their field of expertise than do non-experts. Navigation abilities, for example, have been associated with relative increase in posterior hippocampus grey matter volume accompanied by relative decrease in anterior hippocampus in London taxi drivers. Expert chess players have been found to activate different brain systems than novices [23-27]. In a longitudinal study, Hyde and colleagues found structural brain changes after the relatively short time span of only 15 months of musical training, which were correlated with improvements in musically-relevant motor and auditory skills [28]. By enabling the comparison of experts and non-experts within the auditory domain (musicians versus non-musicians, respectively), musical training has been recognized as an important tool for the investigation of long term training-driven plasticity effects and enhanced auditory processing [29-39]. The processing of sound patterns has also been shown to be improved by long-term musical training [12,13,36,40,41] and tends to be left-lateralized [12,13,40]. It is, however, still unknown how musical experience affects the encoding of patterns that are difficult to extract from an acoustically variable environment.

In the current experiment, we used magnetoencephalography (MEG) to investigate the effect of long-term musical training on the processing of tone patterns that varied in salience within their acoustic context. Note: The term salience or saliency is used throughout this study in its original meaning, that is, the state or condition of being prominent or most noticeable or important, and not in its special meaning in perceptual psychology (i.e. the state or quality by which it stands out relative to its neighbors). We compared musicians’ and non-musicians’ auditory processing in three conditions which differed in the frequency of the standard pattern: salient (70% probability of the standard pattern occurring), less salient (50% probability of the standard pattern) and least salient (35% probability of the standard pattern). In each of these conditions, four deviant patterns with approximately equal probability were presented. The probability of any individual deviant (i.e. deviant pattern) occurring was lower than that of the standard pattern. The deviant and standard patterns were presented randomized as a continuous tone stream. This allowed us to test the following: at which probabilities of regular tone pattern (“standards”) could MMN responses to violations of this pattern (“deviants”) be observed at all (i.e. the lower threshold of standard probability); how the MMN response is affected by standard probability in tone patterns; and how musical expertise affects the encoding of the tone patterns. Additionally, a standard frequency oddball condition was recorded in which no differences between the groups were expected.

## Results

### MEG data

Amplitudes and latencies of the MMN were entered into statistical analyses. In all analyses the alpha level was 0.05 and tests were two-tailed.

### Effect of standard probability and hemisphere on the MMN

**Figure 1:**
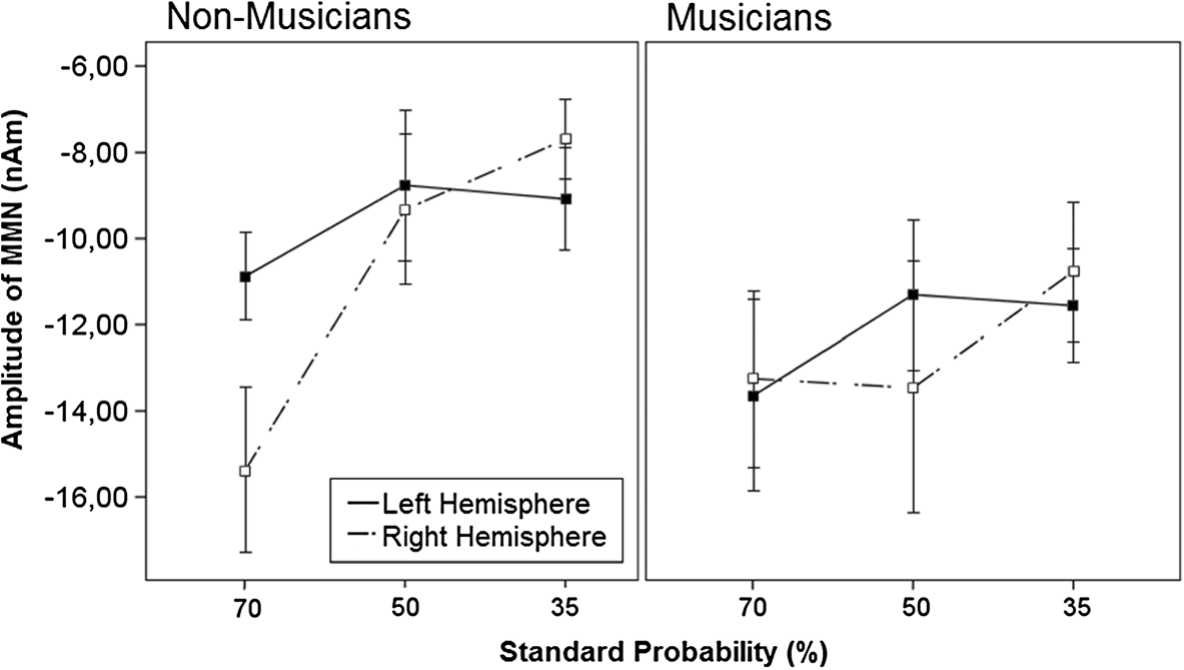
**Grand averaged source waveforms of the three conditions separated by group and hemisphere.** The 35% standard probability condition is shown in the upper row, the 50% standard probability condition in the middle row and the 70% standard probability condition in the lower row. The grand averaged source waveforms (solid lines) obtained from the individual dipole moment of MMN for musicians (**A**) and non-musicians (**B**) are presented with 95% bootstrapped confidence intervals (gray shaded areas). Time windows in which the 95% confidence interval of the bootstrap around the averaged source waveform did not include zero values were considered to indicate significant deflections. For both groups, the left hemisphere is presented on the left side and the right on the right side. The black triangles indicate the MMN response.

To test in which experimental conditions a significant MMN deflection to the pattern deviants was elicited, we performed nonparametric bootstrapping tests (1000 resampling iterations) on the group averaged waveforms for the MMN in both hemispheres for all three pattern conditions [42-45]. Time windows in which the 0.95 percent confidence interval of the bootstrap did not include zero values were considered to indicate significant deflections. As shown in Figure 1, the MMN deflection is significantly different from zero in all three pattern conditions in both groups.

The amplitudes of the individual MMN difference source waveform peaks of the three pattern condition (pattern deviant) were entered into a mixed model 2 × 2 × 3 ANOVA with the between subject factor being group (musicians and non-musicians) and the within subject factors being hemisphere (left and right) and standard probability (35%, 50% and 70%).

**Figure 2:**
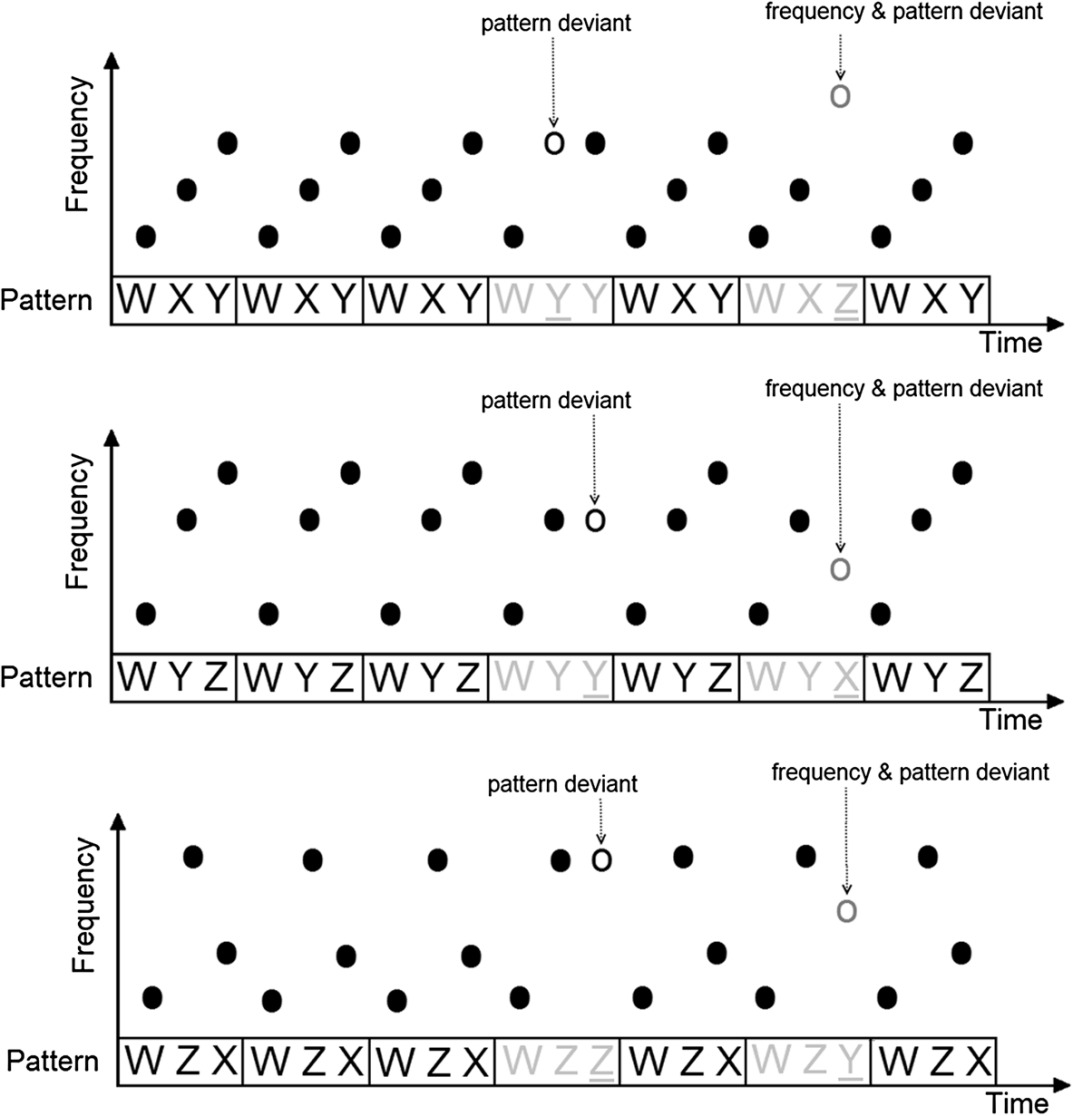
**Interaction effect of hemisphere × standard probability in the two groups with corresponding standard error bars.** The non-musicians are presented at the left panel and the musicians at the right panel. The MMN amplitude of the right hemisphere in the non-musicians is significantly larger in the 70% condition than in the 50% and 35% conditions, whilst the amplitude of the left hemisphere remains stable. In the non-musicians, the MMN amplitude of the right hemisphere is significantly larger than in the left hemisphere in the 70% condition. There are no significant differences between conditions in the group of musicians.

For the MMN amplitude we found a significant main effect of the standard probability [F (2,68) = 3.817, p = .027], and a 2-way-interaction of hemisphere × standard probability [F (2,68) = 4.742, p = .012] as well as a 3-way-interaction of hemisphere × standard probability × group [F (2,68) = 5.331, p = .007]. In order to explore the source of significant interaction we conducted additional ANOVAs: amplitude was entered into repeated-measures 2 × 3 ANOVAs using within subject factors of hemisphere (left and right) and standard probability (35%, 50% and 70%) for each group (musicians and non-musicians). The results showed no significant main effects or interactions within the group of musicians, while the results within the group of non-musicians revealed differences a main effect of standard probability [F (2,34) = 5.050, p = .012]) as well as difference in lateralization of the MMN for specific standard probability (2-way-interaction of hemisphere × standard probability [F (2,34) = 9.550, p = .001]). To understand the effect of the 3-level factor standard probability we conducted 2 repeated-measures 1-way-ANOVAs for the group of non-musicians for each hemisphere (left and right) with the factor standard probability (70%, 50% and 35% standard probability, respectively) and to analyze the effect of hemisphere on MMN amplitude separately for each standard probability, afterwards paired-samples t-tests for non-musicians were conducted to compare the two hemispheres (left and right) in amplitude for every standard probability (70%, 50% and 35% standard probability, respectively). Because both analyses are using the same data the alpha level was divided by two (p < .025) in order to correct for multiple comparison. The results of the 1-way-ANOVAs show a significant effect of standard probability in the right hemisphere [F (2,16) = 7.799, p = .004] with a post-hoc Bonferroni pairwise comparison revealing a more pronounced MMN in the 70% compared to the 50% [mean difference = ±6.071, p = .022] and the 35% standard probability [mean difference = ±7.705, p = .003]There was no significant effect in the left hemisphere. The results of the, paired-samples t-tests for non-musicians show a significant difference in amplitude between left and right hemisphere in the for the 70% standard probability condition [t (1,17) = 2.679, p = .016] and no effects in the other standard probability conditions. The interaction of hemisphere × standard probability in the non-musicians and musicians is depicted separately in Figure 2 and the corresponding waveforms in Figure 1. All results of the pattern conditions are shown in Additional file 1.

The statistical analysis of the pitch oddball condition in a mixed model 2 × 2 ANOVA, with the between subject factor being group and the within subject factor being hemisphere revealed, for amplitude, as expected, no significant differences between groups or hemispheres.

### Behavioural data – questionnaire

Immediately after the test, participants were asked two questions. The first question (Q1) asked if they had observed anything in particular about the auditory stimuli they had heard during the experiment. We expected that an affirmative answer would be followed by the report that they noticed regular tone patterns, which were occasionally interrupted by different tone patterns. The second question (Q2) asked them directly if they had noticed regular patterns and, if they had, in which run they had noticed them first. Three subjects missed to answer Q1: 2 musicians and 1 non-musician. Of the remaining 33 subjects, 11 out of 16 musicians and 7 out of 17 non-musicians reported detecting the presence of the tone patterns. Q2 was answered by 32 subjects (15 musicians and 17 non-musicians), 10 musicians and 6 non-musicians reported to have noticed the pattern structure. The difference in the answers (coded as correct and incorrect) between the groups in both questions, as revealed by an χ^2^-test, was not statistically significant, but there was a trend in the difference in Q2 in favor of the musicians [χ^2^(1) = 3.137, p = .077].

## Discussion

In this study, we have shown how the statistical salience of sound patterns in an acoustic environment and long-term musical expertise affect the auditory encoding of such patterns. Musicians and non-musicians passively listened to three runs of tone pattern streams that consisted of different tone patterns. Within each run, one of the patterns had the highest probability of occurring, thus making it the standard pattern. The probability of this most frequent standard pattern differed between runs (salient condition 70%, less salient condition 50% and least salient condition 35% standard probability). We observed a significant MMN response in all three conditions of acoustic salience, including the least salient condition (which featured a standard probability of 35%), in both groups. The MMN amplitude of the non-musicians was influenced by standard probability and was more pronounced in the salient (70% standard) condition than in the less and least salient (50% and 35% standard) conditions, especially in the right hemisphere. The musicians´ MMN amplitudes, however, were not influenced by standard probability, leading to the interpretation that the lateralized dependency of the MMN amplitude on salience was modulated by the factor long term musical training.

### MMN in an acoustic environment with a low level of salience

In MMN study designs, the standard probability is very often set to an average of 70-90%. For simple feature MMN, approaches such as the multi-features designs [46-49] have shown in an impressive way that MMN can be elicited with 50% standards or even without an actual standard [50]. However, we are not aware of studies that investigated the lower threshold of the standard probability in higher-order pattern processing. In the present study we observed an MMN response to tone pattern violations with a standard probability of only 35% in both musicians and non-musicians. As in the above mentioned multi-feature paradigms also in the present study a certain aspect of the standard representation has been reinforced on a local-regularity-level (single tone). The global-regularity level (pattern) on the other hand which had to be encoded first to understand the violations to the patterns the standard probability was in the lowest condition only 35%. To our knowledge, this is the first demonstration that tone patterns can be encoded as the standard even at such a low level of salience.

This finding is in line with previous research, which has indicated that expectations can be formed on the basis of global characteristics of the auditory environment, such as the present tone patterns, and not only on the basis of local regularities [10-13]. It, furthermore, shows that the comparator mechanism underlying the mismatch negativity of the auditory system is far more sophisticated and sensitive to regularities in the auditory environment, even in the presence of high levels of statistical noise (deviants), than was assumed until now. In order to detect regularity violation, the regularity has first to be established, a process also known as standard formation [51]. The results of the present study suggest that the ability of our auditory system to form a standard representation in more complex designs, such as tone patterns, needs a surprisingly low signal (standards) among the noise (deviants). While other studies [12,13,52] have used standard probabilities of 50%, lower probability rates have not been used. Since, in our paradigm, all conditions yielded significant MMN deflections, further research is necessary to detect the lower boundary of standard probability that allows pattern detection to take place. On the basis of the present results, this lower threshold should be even less than 35%. It would also be interesting to investigate the percentage of standard probability at which deviant detection enters a conscious stage and how it is influenced by musical expertise. This question could not be addressed within the current design but the behavioural data we obtained suggest that this may indeed be influenced by musical training. The focus of attention may also play a role [53,54].

### Hemispheric lateralization of pattern processing

In line with previous studies which used simple feature oddball paradigms [18-20], we found that pattern MMN amplitude varied with standard probability at least in the non-musicians. One possible reason why Sculthorpe and Campbell [17] did not observe this relationship could be ceiling effects due to the different standard probabilities they used. The probabilities they used were much higher than in the present study and the deviants of each of that probabilities were, therefore, able to elicit pronounced MMN responses. In other words, increasing the standard probability over a certain level may not enhance the MMN amplitude any further. By using sufficiently different salience levels we were able to modulate MMN amplitude. The relationship between standard probability and MMN amplitude has been interpreted as evidence of a stronger memory trace due to easier formation of the standard. In a recent study Bendixen & Schröger (2008) showed that the relationship of standard probability and MMN amplitude (or inverse relationship of deviant probability and MMN amplitude) is not observed for abstract regularities [55]. The correlation found in single-feature MMN was attributed by the authors to a contamination of the response by refractoriness. Nevertheless this contamination problem is not part of the design of the present study. This indicates that the explanation that MMN probability effects are due to N1 refractoriness as suggested by Bendixen & Schröger (2008) and reasoned by Sculthorpe and Campbell (2011) may be too simple. Probabilities of abstract patterns modulate MMN amplitude in a way that seems independent of refractoriness.

We were, furthermore, interested in the role of the right and left auditory cortices in tone pattern processing. As can be seen in Figure 1, the standard probability mainly affects the MMN response in the right hemisphere of non-musicians, while responses in the left hemisphere of non-musicians, and in both hemispheres of musicians were not significantly affected by standard probability across the different conditions. Previous work has shown that acoustic stimuli, such as music and tones, are predominantly processed in the right auditory cortices [1,3,56,57]. On closer examination, however, this lateralization seems to be dependent on the particular stimulus parameters involved. The processing of tone patterns, for example, was lateralized towards the left hemisphere in previous MMN studies [12,13,41]. Lateralization of tone processing is also modulated by the rhythmic and metric structure of tone sequences, familiarity, top-down expectations and musical expertise [40,56,58-61]. Processing can even shift from one hemisphere to the other when arbitrary stimuli become meaningful in the course of a short-term training procedure [62]. In the present study, tone patterns were used as stimulus material and, whereas the results did not show an overall lateralization of processing, we found a modulation of the MMN amplitude by the standard probability only in the right hemisphere of non-musicians, while the left hemisphere was not significantly affected by standard probability. This may imply that the processing of tone patterns in a salient environment is right lateralized, but as the level of salience (deviant probability) decreases and the regularities are more difficult to encode, additional processing in the left hemisphere is required.

### Influence of musical expertise

A large body of literature shows that musical experience, in both the long and short term, modulates the processing of auditory material, especially for stimuli such as complex tone patterns [12,13,40,63,64]. In the present study, auditory processing of tone patterns in the musicians was not influenced by the standard probability, but this influence was apparent in the case of the non-musicians. The non-musicians´ right hemispheres were more strongly influenced than the left hemispheres, especially in the 70% standard probability condition. This implies that the auditory processing of musicians is not dependent on the level of salience as used in our study, albeit it could be possible that with even lower standard probabilities an influence on the MMN could be seen also in musicians. Previous research has shown that the processing of more complex material, such as tone patterns, as opposed to the classic oddball paradigm stimuli, is facilitated by musical expertise [12,59,61,65], possibly because formal musical training would direct musicians toward more analytical processing of acoustic stimuli. Although the high standard probability stimuli used in the current study are not as simple as the classic oddball material, we consider them to be simpler than the lower standard probability stimuli (essentially, the lower the standard probability, the higher the complexity) and, as such, the results of the current study fit well with previous research findings regarding musicians’ greater facility for processing of complex auditory stimuli. These superior auditory processing abilities may explain the absence of an effect of standard probability upon the musicians. This demonstration that musicians are less affected by the variability of the signal in the detection of acoustic regularities reflects their expertise in the auditory domain.

## Conclusion

The results indicate that the MMN amplitude in the right hemisphere of non-musicians in response to deviants in tone patterns is influenced by the probability of the occurrence of the standard pattern, with the effect being greater for more salient acoustic stimuli. The amplitude of the MMN response in the left hemisphere of non-musicians is more stable. The MMN response in musicians, on the other hand, did not seem to be influenced by the level of salience. This implies that violation detection processing in non-musicians (ie. comparative non-experts in auditory processing) is dependent on the salience of the acoustic environment: in acoustic environments with a low level of salience, detection of change is more challenging for the auditory networks than in a more salient acoustic environment.

## Methods

### Subjects

21 musicians and 22 non-musicians participated in the experiment. Seven subjects (3 musicians, 4 non-musicians), were excluded from the final analysis due to insufficient MEG recording quality, excessive head movements, or insufficient quality of the model fit of their recorded data (exclusion criteria: the dipoles explained less than 85% of the magnetic field variance) resulting in a total number of 36 subjects (18 musicians, mean age 24.61 (*SD* 2.81), 7 males; 18 non-musicians, mean age 25.5 (*SD* 2.85), 6 males). Musicians were students at the Music Conservatory in Münster or professionals or had received extensive musical training since childhood (minimum ten years) and were still actively playing (average practice time of 18.11 hours per week) as evaluated by a questionnaire; none of them had absolute pitch (self-report). Non-musicians were classified by not having received any musical training apart from basic compulsory music classes in school. All subjects were right-handed as assessed by the Edinburgh Handedness Inventory [66], had normal hearing as assessed by clinical audiometry, and provided written consent prior to their participation in the study. The study protocol was approved by the ethics committee of the Medical Faculty of the University of Münster and the study was conducted according to the Declaration of Helsinki.

### Stimuli and procedure

**Figure 3:**
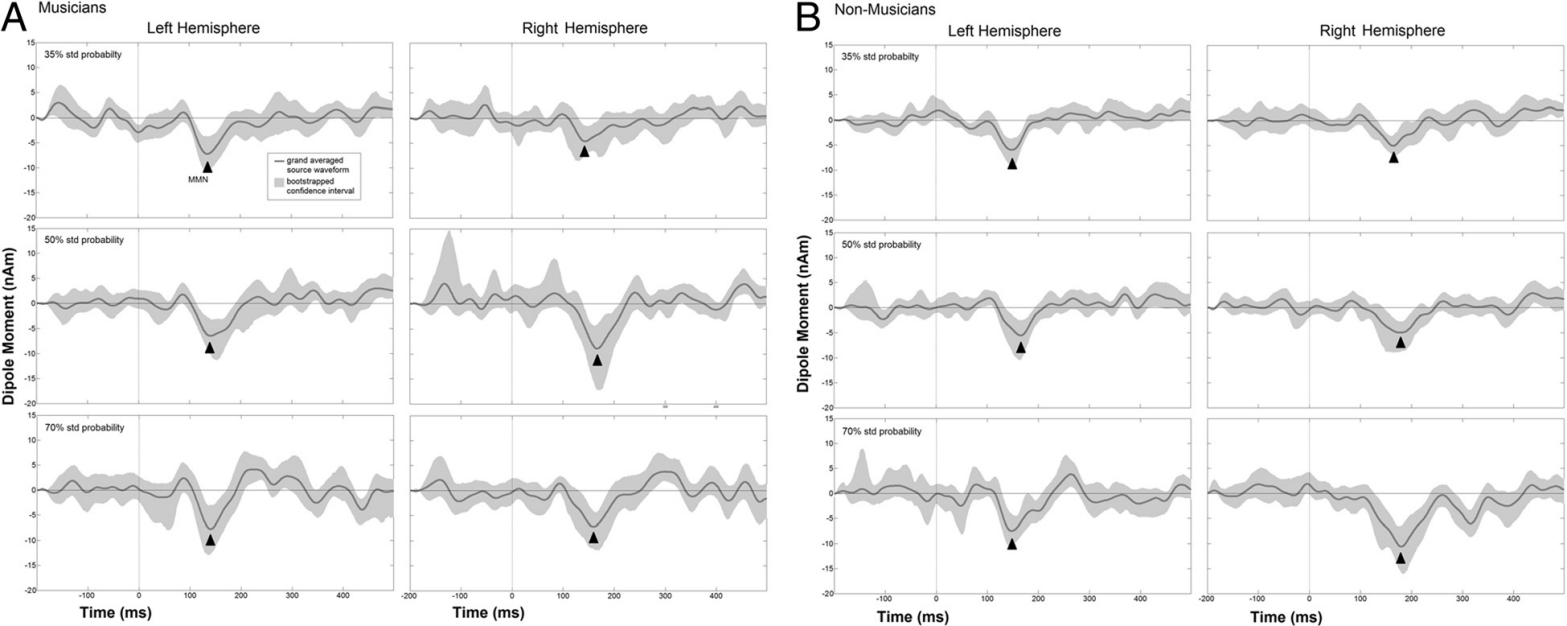
**Outline of the three standards of the pattern condition (standard, pattern deviant, and frequency and pattern deviant).** The patterns were embedded in a continuous sound stream with standard pattern probability set to 35%, 50% and 70%, respectively.

The tones were generated in 44100 Hz stereo and 32 bit, the frequencies used were W = 500 Hz, X = 594 Hz, Y = 705 Hz and Z = 838 Hz, which equate to ascending tones with a pitch difference of three semitones. The duration of each tone was 200 ms including 10 ms rise and decay time, and the interstimulus interval was set to 400 ms. Each trial consisted of three tones forming one tone pattern. Tone patterns always started with tone W. Half of the deviants were pure pattern deviants, in the sense that they contained no tones of a different frequency than the tones of the standard pattern, and the other half were combined frequency and pattern deviants that contained one tone with a different frequency than the tones of the standard pattern. Examples for both types of deviants are depicted in Figure 3. Three different tone patterns were used as standard patterns: WXY (with corresponding deviant patterns WYY, W**Z**Y, WX**Z** and WXX), WYZ (corresponding deviant trials W**X**Z, WZZ, WY**X** and WYY) and WZX (corresponding deviant trials WXX, W**Y**X, WZ**Y** and WZZ). Whilst both pattern (violating only the pattern but not the pitch of the standard stimuli) and combined pattern and frequency (violating the pattern as well as the pitch of the standard stimuli) deviants were analysed, we focused our interpretation on the results for the pure pattern deviants because we were mainly interested in the higher order regularities. The combined pattern and frequency deviants were only included into the stimulus material to increase the number of different deviant patterns rendering any of the deviant patterns less probable than the standard pattern but could not be analyses due to refractoriness confounds introduced by the additional tone frequency which occurs more rarely than any of the other tones in the sequence. The probabilities of the 4 different deviant patterns were the following: 7 to 8% in the 70% standard pattern condition, 10 to 15% in the 50% standard pattern condition and 15 to 20% in the 35% standard pattern condition (with decreasing standard probability the deviant probability was increasing, accordingly). The mismatch between standard and deviant pattern in both deviant types could either be at the second or the third position of the pattern (as indicated by underscore, see above), and each possibility was presented in half of the deviant trials. The presentation of the stimulus material (the 3 different standard pattern conditions and the oddball condition) was divided into 4 runs of approximately 12 minutes each. One out of the three standard probabilities (70% = salient, 50% = less salient and 35% = least salient) was presented in each of the 2nd to 4th) run, and counterbalanced across standard tone pattern and run order amongst the subjects. The tone patterns were presented as a continuous sound stream with no perceivable gaps between consecutive tone patterns. In total, 400 trials were presented for each run of the each of three pattern conditions.

We used a classic frequency oddball paradigm (500 Hz and 530 Hz tones, one semitone difference) as a control condition in which a reliable mismatch response was expected in all subjects. This condition was presented before the three pattern conditions in run 1. The tones were presented as continuous stream with tone duration of 200 ms including 10 ms rise and decay and an ISI of 500 ms. In total, 995 tones were presented. The probability of deviant occurrence was set at 0.15 and at least 3 standards preceded each deviant

Participants passively listened to all conditions while they were attending to a silent movie (Disney´s “Peter Pan”). After each run, they had to answer four to six questions about the content of the movie to ensure that they paid attention. After the last run, participants were debriefed and asked if they noticed anything in particular about the acoustic stimuli. If they did not they were specifically asked if they noticed a pattern in the acoustic stimuli and, if they did, during which run they first noticed it. The overall duration of the experiment was approximately 1.5 hours and the total time of the MEG recordings was approximately 48 minutes.

### MEG recordings

Magnetic fields were recorded with a 275 channel whole-head system (OMEGA, CTF Systems Inc, Port Coquitlam, Canada) in an acoustically and magnetically shielded room. MEG data were acquired continuously during presentation blocks with a sampling rate of 600 Hz. The subjects passively listened to the four blocks (one run of pitch oddball condition, three runs of pattern conditions) with short breaks in between, during which they could relax and were asked questions about the content of the movie by the investigator. Participants were seated upright and their head position was comfortably stabilized with pads inside the dewar. Stimuli were delivered via air conduction through plastic tubes at 60 dB above the individual hearing threshold, which was determined for each ear at the beginning of each MEG session for the different stimuli with an accuracy of 5 dB. The subject’s alertness, well-being and compliance were verified by video monitoring. The subjects were instructed to minimize swallowing and blinking.

### Data analysis

The continuous data were separated into epochs of 600 ms, starting 100 ms before and ending 500 ms after the tone onset of the deviant tone of a deviant pattern, or the corresponding standard tone of a standard pattern (the standard tone at the same position as the deviant tone analyzed) in all 3 pattern conditions (35%, 50%, 70%). In the pitch oddball condition analogous epochs of 600 ms were extracted from all deviants and every second standard before a deviant. Epochs containing signal amplitudes larger than 2.5 pT were considered artifacts and were excluded from averaging. Baseline correction was based on the 100 ms baseline before the tone onset of each epoch. Standards and deviants were averaged separately and digitally filtered (high pass filter of 1 Hz and a low pass filter of 30 Hz). Averaged responses to standards were subtracted from averaged responses to deviants in order to acquire the difference response containing the MMN in all conditions.

In the analysis of the data, two equivalent current dipoles (ECD), one in each hemisphere, were used to model the MMN field, a technique justified by the dipolar distribution of the MMN [67].

The ECDs were fitted simultaneously in a spherical volume conductor to each individual’s peak of MMN (restricted to the predefined MMN window of 120 to 250 ms) in the averaged difference response. Source waveforms for each of the participants in each of the conditions were derived from the MEG data using the technique of signal space projection [68], thereby reducing the data to one source waveform for each hemisphere. MMN sources are assumed to remain relatively stable across similar stimulations and the source space projection method is robust to slight displacements of sources. The fit from the model with the best signal-to-noise-ratio was used for all conditions within one subject. All dipolar sources included in the analysis explained at least 85% of the magnetic field variance with a mean goodness of fit of 90.7% and no significant difference between the groups was found with an independent sample t-test ([T (1,34) = .382, p = .705], mean goodness of fit for NM = 90.87%, M = 90,53%).

## Competing interests

The authors declare that they have no competing interests.

## Authors’ contributions

AK, EP, SCH and CP designed the experiment and interpreted the results; AK carried out data acquisition and analysis and drafted the manuscript; EP, SCH and CP revised the manuscript. All authors read and approved the final manuscript.

## Supplementary Material

Additional file 1 **Is a table showing all results of all analysis performed for the MEG data for the pattern conditions.** The title reads: Results of all analysis performed with the MEG data for the pattern conditions. Significant results are marked with an asterix. Click here for file
