# Supplementary material for: Effects of musical training and event probabilities on encoding of complex tone patterns
Source: BMC Neurosci. 2013 Apr 24;14:51. doi: 10.1186/1471-2202-14-51 (PMC3639196; doi:10.1186/1471-2202-14-51)
Supplement: Additional file 1 — Is a table showing all results of all analysis performed for the MEG data for the pattern conditions. The title reads: Results of all analysis performed with the MEG data for the pattern conditions. Significant results are marked with an asterix. [file 1471-2202-14-51-S1.pdf]

Additional file 1: Results of all analysis performed with the MEG data for the pattern conditions. Significant results are marked with an asterix.

Analysis performed	Effect / Interaction	f-value	t-value	Mean difference	df	p-value
Mixed model 2 × 2 × 3	hemisphere	.787			(1,34)	.381
ANOVA	standard probability	3.817			(2,68)	**.027***
	group	1.697			(1,34)	.201
	hemisphere × standard	4.742			(2,68)	**.012***
	probability	.746			(2,68)	.478
	group × standard	.271			(1,34)	.606
	probability	5.331			(2,68)	**.007***
	Hemisphere × group					
	hemisphere × standard					
	probability × group					
Repeated-measures 2 × 3 ANOVA, group of musicians	Hemisphere	.046			(1,17)	.833
	Standard probability	.604			(2,34)	.552
	hemisphere × standard probability	1.896			(2,34)	.166
Repeated-measures 2 × 3 ANOVA, group of non-musicians	Hemisphere	1,830			(1,17)	.194
	Standard probability	5.050			(2,34)	**.012***
	hemisphere × standard probability	9.550			(2,34)	**.001***
Paired-samples t-tests, left vs. right hemisphere, group of non-musicians	Probability .70		2.679		(1,17)	**.016***
	Probability .50		.624		(1,17)	.541
	Probability .35		-1.641		(1,17)	.119
Repeated-measures 1-way-ANOVA, left hemisphere, group of non-musicians	Standard probability	1.771			(2,16)	.202
Repeated-measures 1-way-ANOVA, right hemisphere, group of non-musicians	Standard probability	7.799			(2,16)	**.004***
Bonferroni pairwise comparison, right hemisphere, group of non-musicians	70 compared to 50			±6.071		**.022***
	70 compared to 35			±7.705		**.003***
	50 compared to 35			±1.634		1.000
